# Population Pharmacokinetics of Tamibarotene in Pediatric and Young Adult Patients with Recurrent or Refractory Solid Tumors

**DOI:** 10.3390/curroncol31110527

**Published:** 2024-11-14

**Authors:** Takuya Azechi, Yutaka Fukaya, Chika Nitani, Junichi Hara, Hiroshi Kawamoto, Tomoaki Taguchi, Kenichi Yoshimura, Akihiro Sato, Naoko Hattori, Toshikazu Ushijima, Toshimi Kimura

**Affiliations:** 1 Department of Pharmacy, Juntendo University Hospital, Bunkyo-ku, Tokyo 113-8431, Japan; t-azechi@juntendo.ac.jp (T.A.);; 2 Faculty of Pharmacy, Juntendo University, Urayasu 279-0013, Chiba, Japan; 3 Department of Pediatric Hematology/Oncology, Osaka City General Hospital, Osaka 534-0021, Osaka, Japan; c-tanaka@med.osakacity-hp.or.jp (C.N.); j-hara@med.osakacity-hp.or.jp (J.H.); 4 Department of Pediatric and Allergy, Fujimi Clinic, Ota-ku, Tokyo 143-0015, Japan; 5 Fukuoka College of Health Sciences, Fukuoka 814-0193, Fukuoka, Japan; 6 Department of Biostatistics and Health Data Science, Nagoya City University Graduate School of Medical Science, Nagoya 467-8601, Aichi, Japan; 7 Clinical Research Support Office, National Cancer Center Hospital East, Kashiwa 277-8577, Chiba, Japan; 8 Department of Epigenomics, Institute for Advanced Life Sciences, Hoshi University, Shinagawa-ku, Tokyo 142-8501, Japan; naoko.hattori@gunma-u.ac.jp (N.H.);

**Keywords:** tamibarotene, population pharmacokinetics, pediatric, young adult

## Abstract

Tamibarotene is a synthetic retinoid that inhibits tumor cell proliferation and promotes differentiation. We previously reported on the safety and tolerability of tamibarotene in patients with recurrent or refractory solid tumors. Therefore, in this study, we aimed to evaluate the pharmacokinetic properties of tamibarotene and construct a precise pharmacokinetic model. We also conducted a non-compartmental analysis and population pharmacokinetic (popPK) analysis based on the results of a phase I study. Targeted pediatric and young adult patients with recurrent or refractory solid tumors were administered tamibarotene at doses of 4, 6, 8, 10, and 12 g/m^2^/day. Serum tamibarotene concentrations were evaluated after administration, and a popPK model was constructed for tamibarotene using Phoenix NLME. During model construction, we considered the influence of various parameters (weight, height, body surface area, and age) as covariates. Notably, 22 participants were included in this study, and 109 samples were analyzed. A two-compartment model incorporating lag time was selected as the base model. In the final model, the body surface area was included as a covariate for apparent total body clearance, the central compartment volume of distribution, and the peripheral compartment volume of distribution. Visual prediction checks and bootstrap analysis confirmed the validity and predictive accuracy of the final model as satisfactory.

## 1. Introduction

Neuroblastoma is one of the most prevalent solid tumors in childhood, following leukemia and brain tumors [1]. Risk stratification for neuroblastoma relies on clinical factors, such as age at diagnosis, disease characteristics, and molecular biology markers, for guiding personalized treatment strategies [2,3]. Five-year survival is over 90% for low-risk patients, while it decreases to less than 50% for high-risk groups [4]. Moreover, recurrence in high-risk neuroblastoma is associated with a poor prognosis, with overall survival dropping to approximately 20% [5]. Consequently, developing effective therapies for high-risk neuroblastoma remains an urgent necessity.

In recent years, the efficacy of administering retinoic acid following myeloablative high-dose chemotherapy has been reported in reducing relapse risk for high-risk neuroblastoma patients [6,7]. Retinoic acid suppresses the proliferation and induces the differentiation of tumor cells by binding to retinoic acid receptors (RAR-α, -β, and -γ) [8,9]. Currently, 13-cis-retinoic acid is utilized off-label for high-risk neuroblastoma treatment in several countries [7]; however, it remains unavailable in Japan, highlighting the need for new therapeutic agents, such as 13-cis-retinoic acid.

Discovered by K Shudo et al., tamibarotene is a synthetic retinoid which selectively binds to RAR-α and -β [10,11,12]. Tamibarotene induces the differentiation of leukemia cells; therefore, it can effectively treat acute promyelocytic leukemia in newly diagnosed patients and those who relapse after treatment with all-trans-retinoic acid and conventional chemotherapy in Japan [13,14,15]. In addition, the results of a phase II study indicated its usefulness in treating advanced hepatocellular carcinoma, a solid tumor [16]. Furthermore, tamibarotene induces differentiation in neuroblastoma cell lines [17,18]. Additionally, the efficacy of tamibarotene combined with decitabine (5-aza-dC) in treating neuroblastoma was reported in an in vitro study [19]. Therefore, there is an urgent need to evaluate the dose, efficacy, and safety of tamibarotene in patients with neuroblastoma. In adult pharmacokinetics, tamibarotene exhibits dose-dependent relationships in peak plasma concentration and area under the curve (AUC) [16,20]. However, no pharmacokinetic analysis has been conducted specifically for pediatric patients. We previously reported on the safety and tolerability of tamibarotene in pediatric and young adult populations in a phase I study [21]. Nevertheless, no studies have examined the population pharmacokinetic (popPK) profile or covariate factors influencing tamibarotene pharmacokinetics in pediatric patients. Therefore, elucidating the pharmacokinetic details and identifying contributing factors remain essential to ensure the safe use of tamibarotene in pediatric patients.

Therefore, in this study, we performed non-compartmental analysis (NCA) and popPK analysis to evaluate the pharmacokinetic characteristics and construct a popPK model of tamibarotene in pediatric and young adult patients.

## 2. Materials and Methods

### 2.1. Participants and Study Design

In this study, we selected patients aged 3–30 years, diagnosed with advanced or recurrent solid tumors, including histologically confirmed sarcomas, blastomas, germ cell tumors, and CNS tumors, except malignant lymphoma. We planned a phase I, open-label, multicenter, 3 + 3 dose-escalation study conducted at four medical institutions in Japan. The investigational agent, tamibarotene, was orally administered at doses of 4, 6, 8, and 10 mg/m^2^/day and divided into two daily doses. In this study, we utilized a soft capsule formulation containing 1 mg of tamibarotene per capsule, suitable for oral administration in pediatric patients. This study commenced with a 2-on, 2-off dosing schedule. However, this was subsequently modified to a 3-on, 1-off regimen, with 12 mg/m^2^/day established as the maximum dose after no adverse events were observed. Ethical approval for this study was obtained from all medical institutions. This study was explained to the research participants, and they provided informed consent, showing their willingness to participate. This phase I study was registered with the UMIN Clinical Trials Registry (UMIN-CTR, identifier: UMIN000017053).

### 2.2. Sample Collection and Extraction

For the pharmacokinetic analysis, blood samples for the first dose were collected on day 1 immediately before administration and 2, 4, 8, and 10 h post-administration. Blood sampling was performed before the initial oral intake on day 14 (before breakfast). After drawing blood, the samples were stored at <4 °C, followed by centrifugation within 15 min post-collection (1500× *g* for 10 min at 4 °C), and the plasma obtained was aliquoted into serum sample tubes. Subsequently, the samples intended for analysis were frozen and stored at −80 °C under light-protected conditions.

### 2.3. Determination of Serum Tamibarotene Levels

After thawing the frozen plasma samples at room temperature, an equal volume of acetonitrile was added to 25 μL of sample. The mixture was vortexed, left undisturbed at −40–−20 °C for 15 min, and then subjected to centrifugation at 500× *g* for 5 min at 4 °C. The supernatant was used as the sample for measurement. Serum tamibarotene concentrations were evaluated using liquid chromatography/tandem mass spectrometry, developed and validated at the Shimadzu Techno-Research Laboratory (Japan). The separation of tamibarotene was conducted using the high-performance liquid chromatography system CBM-20A, comprising an analysis column (Unison UK-C18 [Imtakt Corporation, Kyoto, Japan], 250 mm × 3 mm, 3 μm), a degassing system, a delivery unit, a thermostat-controlled autosampler and column compartment, and a diode array detector. Mobile phase A comprised water/acetonitrile/acetic acid (90:10:0.1), and mobile phase B contained acetonitrile/2-propanol/acetic acid (50:50:0.1). The column flow rate was set at 0.4 mL/min, with an injection volume of 5 μL. The column temperature was maintained at 50 °C, and the autosampler temperature was set at 10 °C. The conditions for Q1 (precursor ion) and Q3 (product ion) in multiple reaction monitoring were set as follows: Q1 = 352.1 *m*/*z* and Q3 = 149.0 *m*/*z* for tamibarotene and Q1 = 358.1 *m*/*z* and Q3 = 149.0 *m*/*z* for tamibarotene international standard (IS: tamibarotene-^13^C_6_).

### 2.4. Method Validation

Tamibarotene and IS were weighed and dissolved in methanol to prepare stock solutions of 1 mg/mL. The stock solution was serially diluted to prepare working solutions of tamibarotene. To prepare plasma calibration samples, working solutions were added to blank human plasma at concentrations of 0.5, 1, 5, 10, 50, 100, and 250 ng/mL. The concentrations of tamibarotene were set at 1.5, 25, and 200 ng/mL for the low-, mid-, and high-concentration quality control (QC), respectively. Linearity was evaluated by plotting the peak area of each calibration sample against the corresponding to tamibarotene concentrations. The calibration curve was analyzed using linear regression with a weighting factor of 1/concentration. The relative error percentage (RE) at each concentration of calibration samples was calculated based on theoretical values using the following formula:

$$RE\;\left(\%\right)=\frac{Measured\;value-Theoretical\;value}{Theoretical\;value}\times 100$$

The Lower Limit of Quantification (LLOQ) was set at 0.5 ng/mL, with an acceptable range for RE at each concentration within ±15%, except for the LLOQ, which was within 20%. In this study, the RE of tamibarotene was −2.2 to 4.6% at the LLOQ and −2.2 to 3.0% at other concentrations, which were within the acceptance criteria. Similarly, the RE of QC samples was −2.4 to 12.0%, which was within the acceptance criteria.

Carryover was assessed by injecting a blank sample after the upper limit of quantification. In this study, the carryovers of tamibarotene and IS were confirmed to be less than 0.1%, which is within the acceptable range.

Additionally, the deviation between the initial measurement and the Incurred Sample Reanalysis (ISR) was 0–17.6%, confirming adherence to the specified acceptance criteria.

### 2.5. Non-Compartmental Model Analysis

Pharmacokinetic parameters were calculated from the data of individual participants through NCA using Phoenix WinNonlin7.0 (Pharsight Corp., Mountain View, CA, USA), a computer program developed for pharmacokinetic (PK) analysis. The PK parameters included the time to reach maximum concentration (T_max_), elimination half-life (t_1/2_), apparent volume of distribution (Vd/F), and apparent total body clearance (CL/F). When calculating the pharmacokinetic parameters, values less than the LLOQ were considered as 0.

### 2.6. The Development of a Tamibarotene popPK Model

PopPK analyses of all concentration/time and patient physiological data were performed using Phoenix NLME 7.0 (Pharsight Corp.). The first-order conditional estimation-extended least-squares method was used to estimate the tamibarotene popPK parameters and variability.

First, one- and two-compartment models with the first-order absorption of tamibarotene, with and without lag time (T_lag_), were tested as structural models. Additive and proportional (exponential) error models were compared for inter-individual and residual variability.

A proportional error model was used to describe the residual variability, which is expressed as follows:

$$C_{obs,ij}=C_{pred,ij}\times \left(1+\epsilon_{ij}\right)$$

where $C_{obs,ij}$ and $C_{pred,ij}$ are the jth observed and predicted plasma concentration for the ith subject, respectively, and $\epsilon_{ij}$ is a random intra-individual error which is normally distributed with a mean of zero and variance of $\sigma^{2}$.

An exponential model was selected to describe the inter-individual variability for all PK parameters, as follows:

$$\theta_{i}=tv\theta \times \exp\left(\eta_{i}\right)$$

where $\theta_{i}$ is the fixed-effect parameter for the ith subject, tvθ is the typical value of the fixed-effect parameter in the population, and $\eta_{i}$ is a random inter-individual variable which is normally distributed with a mean of zero and a variance of $\omega^{2}$.

In the fitting process, participant-specific characteristics such as body weight, height, body surface area (BSA), and age were evaluated for their significance as covariates in the population model to explain the inter-individual variability observed in the PK parameters. The candidate covariate was screened through the simultaneous incorporation of an allometric function on the typical value of clearance (tvCL/F), the typical value of distribution volume (tvV/F), and the mean PK parameters. These are expressed as follows:

CL/Ftamibarotene=tvCL/Ftamibarotens×covariate ^ θ

V/Ftamibarotene=tvV/Ftamibarotene×covariate ^ θ

The final model was determined, according to a previous report [22]. In the present study, changes in the objective function value (OFV; the negative value of twice the log-likelihood difference: −2 LLD) > 6.635 indicate a statistically significant (*p* < 0.01) improvement in the fit of the data.

### 2.7. The Evaluation of a Tamibarotene popPK Model

General goodness-of-fit (GOF) plots and a visual predictive check (VPC) were performed to evaluate the final popPK model of tamibarotene, according to a previous report [22]. GOF plots included predicted plasma concentrations (PRED) based on the mean popPK parameters, individual PRED (IPRED) using Bayesian estimation versus observed concentration, and conditional weighted residual (CWRES) versus the relative elapsed time after dose. In addition, a bootstrap evaluation of the final model was performed using Phoenix 64 NLME 7.0 (with 1000 samples).

## 3. Results

### 3.1. Non-Compartmental Analysis

**Figure 1:**
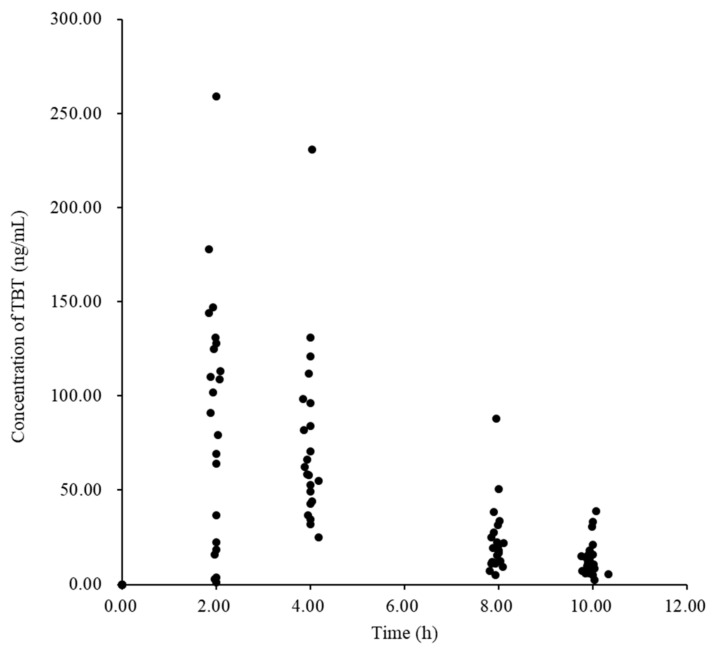
Plot of plasma tamibarotene concentration versus time profile in phase I patients.

Table 1 presents the patient demographic characteristics, and Figure 1 presents the scatter plots of tamibarotene concentration versus time. Data from 21 patients, excluding 1 patient who showed an abnormally high value of T_1/2_ when compared to the others, were analyzed for the single-dose PK analysis. Table 2 shows the PK parameters calculated using NCA.

### 3.2. Tamibarotene popPK Analysis

Notably, 109 samples from 22 patients were used to develop the popPK model. The two-compartment model showed an improvement in the OFV when compared with the one-compartment model. Consequently, the OFV further decreased after adding T_lag_ to the two-compartment model (base model) (Table 3). Compared with the base model (AIC = 949.6917), BSA and body weight as covariates on CL/F significantly decreased the AIC (BSA: 936.411, body weight: 934.464) in the covariate model building with the univariate process. In the final model, BSA was identified as a covariate of CL/F, the central compartment volume of distribution (V_1_/F), and the peripheral compartment volume of distribution (V_2_/F) for tamibarotene in the present study. The final popPK parameters of tamibarotene are listed in Table 4.

### 3.3. Model Validation

**Figure 2:**
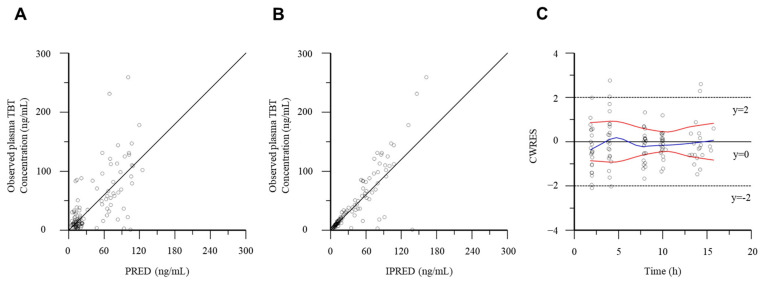
Tamibarotene popPK final model goodness-of-fit plots. (**A**) Observed plasma concentration of tamibarotene vs. predicted plasma concentration (PRED). (**B**) Observed plasma concentrations of tamibarotene vs. individual predicted plasma concentrations (IPRED). (**C**) CWRES (conditional weighted residuals) vs. time.

**Figure 3:**
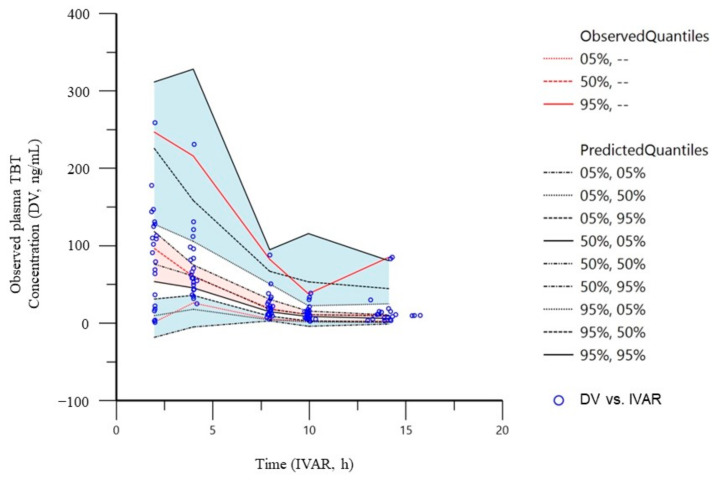
A visual predictive check of the tamibarotene popPK final model. The quantile deviation (blue shaded) obtained from 1000 datasets using the final model was superimposed on the observed tamibarotene concentration of quantile deviation (red shaded). popPK, population pharmacokinetics.

The goodness-of-fit of the final model for the tamibarotene popPK in patients with advanced or recurrent solid tumors was evaluated using PRED, IPRED, and CWRES (Figure 2). Notably, all distributions in the plot of the observed concentrations against PRED or IPRED showed bilateral symmetry around the regression line y = x. The tamibarotene CWRES in the present study was within the range (−3 of 3). The VPC plots of tamibarotene demonstrate that most of the observed concentrations were within the 95% prediction intervals of the simulations, and the median lines of the observed and predicted concentrations were similar (Figure 3). Furthermore, all PK parameters of the final model were within the confidence interval obtained from the bootstrap evaluation, indicating the good predictive performance of the final model (Table 4).

## 4. Discussion

This is the first study to present the popPK analysis results of tamibarotene in pediatric patients in Japan. Previous domestic PK trials lacked popPK analysis, leaving the pharmacokinetic characteristics uncertain; however, in this study, we identified the significance of a two-compartment model. Due to disparity in the models, comparing the distribution volume is challenging; therefore, the clearance in the final model was used to approximate the values obtained from NCA, supporting the validity of the predictive nature of the two-compartment model.

The Tmax value for tamibarotene ranged from 2.26 to 4 h in the NCA, suggesting the prompt absorption of tamibarotene after oral administration. However, while constructing the popPK model, the estimated value of Tlag after adding covariates was approximately 1.8 h, and its physiological validity was considered as low. Therefore, in this study, the final model was built by fixing Tlag to the estimated value before including the covariates.

Generally, with pediatric covariates, the inclusion of parameters in the popPK model may be hindered by the collinearity of parameters associated with body size [23]. Consequently, a single parameter associated with body size was selected for inclusion in this study. Concerning the covariate for the distribution volume (V1/F), the univariate analysis revealed significant fluctuations in CL/F and V1/F for all candidate covariates, suggesting the low physiological validity of the estimated values. Therefore, in this study, within the model incorporating a fixed Tlag, the covariates weight and BSA, which significantly reduced the AIC, were introduced into CL/F before exploring the covariates for V1/F. The BSA utilized in the dosage setting of tamibarotene was the covariate for which all PK parameters were estimated, thereby making it a candidate for the final model.

Furthermore, in the investigation of covariates for V2/F, as all PK parameters were not estimated for any candidate covariate, eliminating the largest η-shrinkage for Ka allowed for the estimation of all PK parameters, enabling the construction of the full model. In the reduced model, excluding BSA as a covariate for V2/F did not change the AIC (full model: 934.146; reduced model: 935.149). This was presumed to be due to the small variation in BSA when compared with the variability in V2/F. However, considering the larger magnitude of V2/F when compared with V1/F and the potential for errors in fixing V2/F, given the developmental process in children, V2/F was retained in the final model. The evaluation of the validity of the results of the VPC and bootstrapping for the constructed final model was considered satisfactory.

This study has some limitations. Notably, the fixed value in this study was used for inter-compartment clearance Q, introducing a potential source of unpredictable errors. Additionally, the limited sample size used in the analysis hindered the attainment of stable analytical outcomes. Consequently, the construction of a full model incorporating all the significant covariates proved challenging. Therefore, increasing the sample size might increase the likelihood of constructing a more precise model. This study was conducted to evaluate the safety and dose of tamibarotene, including patients with malignancies other than neuroblastoma. Consequently, due to differences in tumor location and type, it is plausible that localized or systemic hemodynamics may vary [24,25]. Conversely, there have been reports indicating minimal pharmacokinetic differences across tumor types [26,27]. Future studies should conduct pharmacokinetic analyses of tamibarotene focusing on specific tumor types. Additionally, as the age range of participants in this study spans from 4 to 23 years, it is crucial to consider the potential variability in hepatic clearance as the liver mass and body size undergo significant changes during growth. Tamibarotene is primarily metabolized via hepatic CYP3A4 enzymes; thus, age-related effects on its metabolism cannot be ruled out, even though age was not included as a covariate in the final model. Further pharmacokinetic analysis in specific age populations is warranted.

## 5. Conclusions

We developed a popPK model for tamibarotene in pediatric and young adult patients with recurrent or refractory solid tumors using Phoenix 64 NLME. Our final model showed the significant influence of BSA on the clearance and distribution of tamibarotene. The validity, robustness, and predictability of the final model were established using goodness-of-fit plots, VPC, and bootstrapping. Therefore, the developed model may be useful for future studies on the PK analysis of tamibarotene. These findings, integrated with data on the safety and efficacy of tamibarotene, are anticipated to be beneficial in designing recommended dosage regimens for pediatric use. Furthermore, these findings contribute to a more comprehensive understanding of the pharmacokinetic characteristics of retinoid-based formulations, including tamibarotene, in pediatric patients.

## Figures and Tables

**Table 1:** The demographic data of the patients.

Characteristics	Number	Percentage
Male	15	68.2
Female	7	32.8
Value (units)	Median (Range)	Mean (Standard deviation)
Age (years)	8 (4–23)	9.8 (5.7)
Body weight (kg)	19.9 (14.5–76.6)	28.4 (17.2)
Height (cm)	119 (96.3–185.7)	129.1 (26.9)
Body surface area (m^2^)	0.805 (0.63–1.97)	0.995 (0.389)

**Table 2:** Pharmacokinetic parameters of tamibarotene using non-compartmental analysis.

Patient ID	Kel (/h)	T_1/2_ (h)	Tmax (h)	Cmax (ng/mL)	AUC_0–10_ (ng·h/mL)	AUC_0–∞_ (ng·h/mL)	Vz/F (L)	CL/F (L/h)
1	0.372	1.86	4.00	83.9	330.75	353.91	22.80	8.48
2	0.022	31.24	4.00	34.7	235.56	1610.14	55.98	1.24
3	0.249	2.78	4.00	70.8	366.98	430.41	55.96	13.94
4	0.328	2.12	2.00	69.1	363.40	393.34	62.09	20.34
5	0.229	3.03	4.00	131.0	589.23	735.04	23.76	5.44
6	0.364	1.90	2.08	113.0	363.96	377.94	36.33	13.23
7	0.426	1.63	1.98	131.0	336.34	342.28	27.41	11.69
8	0.275	2.52	2.00	259.0	934.41	1011.82	21.55	5.93
9	0.266	2.60	2.00	64.0	327.68	365.64	30.83	8.20
10	0.418	1.66	4.00	121.0	404.30	429.41	33.42	13.97
11	0.379	1.83	1.92	147.0	463.29	477.30	33.17	12.57
12	0.326	2.13	1.83	144.0	628.71	675.02	13.63	4.44
13	0.253	2.74	1.93	125.0	473.06	524.90	30.16	7.62
14	0.265	2.61	2.00	128.0	331.59	353.30	42.68	11.32
15	0.207	3.36	4.00	52.7	271.91	340.17	56.93	11.76
16	0.200	3.46	2.07	109.0	455.60	544.89	54.93	11.01
17	0.424	1.63	1.87	91.1	418.53	435.19	21.66	9.19
18	0.289	2.40	4.03	231.0	945.23	1079.41	12.82	3.71
19	0.361	1.92	1.83	178.0	658.83	690.65	20.03	7.24
20	0.270	2.56	1.92	102.0	330.45	358.23	92.92	25.12
21	0.257	2.70	1.87	110.0	425.88	486.58	31.99	8.22
22	0.339	2.04	2.03	79.4	305.67	323.33	36.47	12.37
Average *	0.309	2.356	2.541	120.952	463.133	510.893	36.263	10.752
Minimum *	0.200	1.626	1.830	52.700	271.907	323.332	12.815	3.706
Maximum *	0.426	3.458	4.030	259.000	945.232	1079.409	92.924	25.124
SD *	0.071	0.547	0.951	51.808	189.786	214.906	19.179	5.083

* Calculated excluding ID2. AUC, area under the curve; T_1/2_, elimination half-life; Tmax, time to maximum concentration; Cmax, maximum concentration; CL/F, apparent total body clearance; Vz/F, volume of distribution; Kel, elimination rate constant; SD, standard deviation.

**Table 3:** Selection analysis of structure models.

	−2(LL)	ΔOFV	AIC	*p* Value
One-compartment model	984.020	-	998.020	-
Two-compartment model	927.692	56.328	949.692	<0.001
Two-compartment model with *Tlag*	911.838	15.854	937.838	<0.001

−2(LL), twice the log-likelihood difference; AIC, Akaike’s information criterion; OFV, objective function value; *Tlag*, lag time.

**Table 4:** The final model and bootstrap validation.

Final Model	Parameters	Original Estimate	Data (95% CI)	Bootstrap Median	Estimates (95% CI)
CL/F (L/h) = tvCL/F × BSA/mean	tvCL/F (L/h)	8.73	7.12–10.35	9.1	7.61–10.81
Q/F (L/h) = tvQ/F	tvQ/F (L/h)	3.45	1.25–5.65	3.39	2.89–4.70
V1/F (L) = tvV1/F × BSA/mean	tvV1/F (L)	9.17	1.84–16.50	10.13	4.47–15.40
V2/F (L) = tvV2/F × BSA/mean	tvV2/F (L)	60.28	11.10–109.47	48.64	28.67–68.94
Tlag (h)	tvTlag (h)	0.95 (Fixed)	-	-	-
Ka (/h)	tvKa (/h)	0.415	0.270–0.560	0.429	0.350–0.546
Residual variability (%)	-	42.4	-	-	-

Ka, absorption rate constant; BSA, body surface area; CI, confidence interval; Q/F, inter-compartment clearance; V1/F, central compartment volume of distribution; V2/F, peripheral compartment volume of distribution.

## Data Availability

The original contributions presented in the study are included in this article; further inquiries can be directed to the corresponding author.

## References

[1] Nakata K., Matsuda T., Hori M., Sugiyama H., Tabuchi K., Miyashiro I., Matsumoto K., Yoneda A., Takita J., Shimizu C. (2023). Cancer incidence and type of treatment hospital among children, adolescents, and young adults in Japan, 2016–2018. Cancer Sci..

[2] Brodeur G.M. (2003). Neuroblastoma: Biological insights into a clinical enigma. Nat. Rev. Cancer.

[3] Mueller S., Matthay K.K. (2009). Neuroblastoma: Biology and staging. Curr. Oncol. Rep..

[4] Whittle S.B., Smith V., Doherty E., Zhao S., McCarty S., Zage P.E. (2017). Overview and recent advances in the treatment of neuroblastoma. Expert. Rev. Anticancer Ther..

[5] London W.B., Castel V., Monclair T., Ambros P.F., Pearson A.D.J., Cohn S.L., Berthold F., Nakagawara A., Ladenstein R.L., Iehara T. (2011). Clinical and biologic features predictive of survival after relapse of neuroblastoma: A report from the International Neuroblastoma Risk Group project. J. Clin. Oncol..

[6] Matthay K.K., Villablanca J.G., Seeger R.C., Stram D.O., Harris R.E., Ramsay N.K., Swift P., Shimada H., Black C.T., Brodeur G.M. (1999). Treatment of high-risk neuroblastoma with intensive chemotherapy, radiotherapy, autologous bone marrow transplantation, and 13-cis-retinoic acid. Children’s Cancer Group. N. Engl. J. Med..

[7] Matthay K.K., Reynolds C.P., Seeger R.C., Shimada H., Adkins E.S., Haas-Kogan D., Gerbing R.B., London W.B., Villablanca J.G. (2009). Long-term results for children with high-risk neuroblastoma treated on a randomized trial of myeloablative therapy followed by 13-cis-retinoic acid: A children’s oncology group study. J. Clin. Oncol..

[8] Ablain J., de Thé H. (2014). Retinoic acid signaling in cancer: The parable of acute promyelocytic leukemia. Int. J. Cancer.

[9] di Masi A., Leboffe L., De Marinis E., Pagano F., Cicconi L., Rochette-Egly C., Lo-Coco F., Ascenzi P., Nervi C. (2015). Retinoic acid receptors: From molecular mechanisms to cancer therapy. Mol. Asp. Med..

[10] Hashimoto Y., Kagechika H., Kawachi E., Shudo K. (1988). Specific uptake of retinoids into human promyelocytic leukemia cells HL-60 by retinoid-specific binding protein: Possibly the true retinoid receptor. Jpn. J. Cancer Res..

[11] Kagechika H., Kawachi E., Hashimoto Y., Himi T., Shudo K. (1988). Retinobenzoic acids. 1. Structure-activity relationships of aromatic amides with retinoidal activity. J. Med. Chem..

[12] Miwako I., Kagechika H. (2007). Tamibarotene. Drugs Today.

[13] Sanford D., Lo-Coco F., Sanz M.A., Di Bona E., Coutre S., Altman J.K., Wetzler M., Allen S.L., Ravandi F., Kantarjian H. (2015). Tamibarotene in patients with acute promyelocytic leukaemia relapsing after treatment with all-trans retinoic acid and arsenic trioxide. Br. J. Haematol..

[14] Shinagawa K., Yanada M., Sakura T., Ueda Y., Sawa M., Miyatake J., Dobashi N., Kojima M., Hatta Y., Emi N. (2014). Tamibarotene as maintenance therapy for acute promyelocytic leukemia: Results from a randomized controlled trial. J. Clin. Oncol..

[15] Takeshita A., Asou N., Atsuta Y., Sakura T., Ueda Y., Sawa M., Dobashi N., Taniguchi Y., Suzuki R., Nakagawa M. (2019). Tamibarotene maintenance improved relapse-free survival of acute promyelocytic leukemia: A final result of prospective, randomized, JALSG-APL204 study. Leukemia.

[16] Kanai F., Obi S., Fujiyama S., Shiina S., Tamai H., Mochizuki H., Koike Y., Imamura J., Yamaguchi T., Saida I. (2014). An open-label phase I/II study of tamibarotene in patients with advanced hepatocellular carcinoma. Hepatol. Int..

[17] Shiohira H., Kitaoka A., Shirasawa H., Enjoji M., Nakashima M. (2010). Am80 induces neuronal differentiation in a human neuroblastoma NH-12 cell line. Int. J. Mol. Med..

[18] Shiohira H., Kitaoka A., Enjoji M., Uno T., Nakashima M. (2012). Am80 induces neuronal differentiation via increased tropomyosin-related kinase B expression in a human neuroblastoma SH-SY5Y cell line. Biomed. Res..

[19] Hattori N., Asada K., Miyajima N., Mori A., Nakanishi Y., Kimura K., Wakabayashi M., Takeshima H., Nitani C., Hara J. (2021). Combination of a synthetic retinoid and a DNA demethylating agent induced differentiation of neuroblastoma through retinoic acid signal reprogramming. Br. J. Cancer.

[20] Package Inserts of Tamibarotene. https://www.pmda.go.jp/PmdaSearch/iyakuDetail/ResultDataSetPDF/480114_4291014F1021_1_14..

[21] Nitani C., Hara J., Kawamoto H., Taguchi T., Kimura T., Yoshimura K., Hamada A., Kitano S., Hattori N., Ushijima T. (2021). Phase I study of tamibarotene monotherapy in pediatric and young adult patients with recurrent/refractory solid tumors. Cancer Chemother. Pharmacol..

[22] Fukaya Y., Kimura T., Hamada Y., Yoshimura K., Hiraga H., Yuza Y., Ogawa A., Hara J., Koh K., Kikuta A. (2023). Development of a population pharmacokinetics and pharmacodynamics model of glucarpidase rescue treatment after high-dose methotrexate therapy. Front. Oncol..

[23] Meibohm B., Läer S., Panetta J.C., Barrett J.S. (2005). Population pharmacokinetic studies in pediatrics: Issues in design and analysis. AAPS J..

[24] Lugano R., Ramachandran M., Dimberg A. (2020). Tumor angiogenesis: Causes, consequences, challenges and opportunities. Cell. Mol. Life Sci..

[25] Thurber G.M., Weissleder R. (2011). A systems approach for tumor pharmacokinetics. PLoS ONE.

[26] Tortorici M.A., Cohen E.E.W., Pithavala Y.K., Garrett M., Ruiz-Garcia A., Kim S., Fruehauf J.P. (2014). Pharmacokinetics of single-agent axitinib across multiple solid tumor types. Cancer Chemother. Pharmacol..

[27] Biesdorf C., Guan X., Siddani S.R., Hoffman D., Boehm N., Medeiros B.C., Doi T., de Jonge M., Rasco D., Menon R.M. (2024). Pharmacokinetics and immunogenicity of eftozanermin alfa in subjects with previously-treated solid tumors or hematologic malignancies: Results from a phase 1 first-in-human study. Cancer Chemother. Pharmacol..

